# Resonant Raman scattering based approaches for the quantitative assessment of nanometric ZnMgO layers in high efficiency chalcogenide solar cells

**DOI:** 10.1038/s41598-017-01381-4

**Published:** 2017-04-25

**Authors:** Maxim Guc, Dimitrios Hariskos, Lorenzo Calvo-Barrio, Philip Jackson, Florian Oliva, Paul Pistor, Alejandro Perez-Rodriguez, Victor Izquierdo-Roca

**Affiliations:** 1 Catalonia Institute for Energy Research (IREC), Jardins de les Dones de Negre 1, 08930 Sant Adria de Besos, Barcelona Spain; 2 Zentrum fur Sonnenenergie- und Wasserstoff-Forschung Baden-Württemberg (ZSW), Stuttgart, Germany; 3 Centres Cientifics i Tecnologics CCiTUB, Universitat de Barcelona, C. Lluis Sole I Sabares 1, 08028 Barcelona, Spain; 4 Institute of Applied Physics, Academy of Sciences of Moldova, Academiei 5, Chisinau, MD 2028 Moldova; 5 INUB, Departament d’Electronica, Universitat de Barcelona, C. Martı i Franques 1, 08028 Barcelona, Spain

## Abstract

This work reports a detailed resonant Raman scattering analysis of ZnMgO solid solution nanometric layers that are being developed for high efficiency chalcogenide solar cells. This includes layers with thicknesses below 100 nm and compositions corresponding to Zn/(Zn + Mg) content rations in the range between 0% and 30%. The vibrational characterization of the layers grown with different compositions and thicknesses has allowed deepening in the knowledge of the sensitivity of the different Raman spectral features on the characteristics of the layers, corroborating the viability of resonant Raman scattering based techniques for their non-destructive quantitative assessment. This has included a deeper analysis of different experimental approaches for the quantitative assessment of the layer thickness, based on (a) the analysis of the intensity of the ZnMgO main Raman peak; (b) the evaluation of the changes of the intensity of the main Raman peak from the subjacent layer located below the ZnMgO one; and (c) the study of the changes in the relative intensity of the first to second/third order ZnMgO peaks. In all these cases, the implications related to the presence of quantum confinement effects in the nanocrystalline layers grown with different thicknesses have been discussed and evaluated.

## Introduction

**Figure 1:**
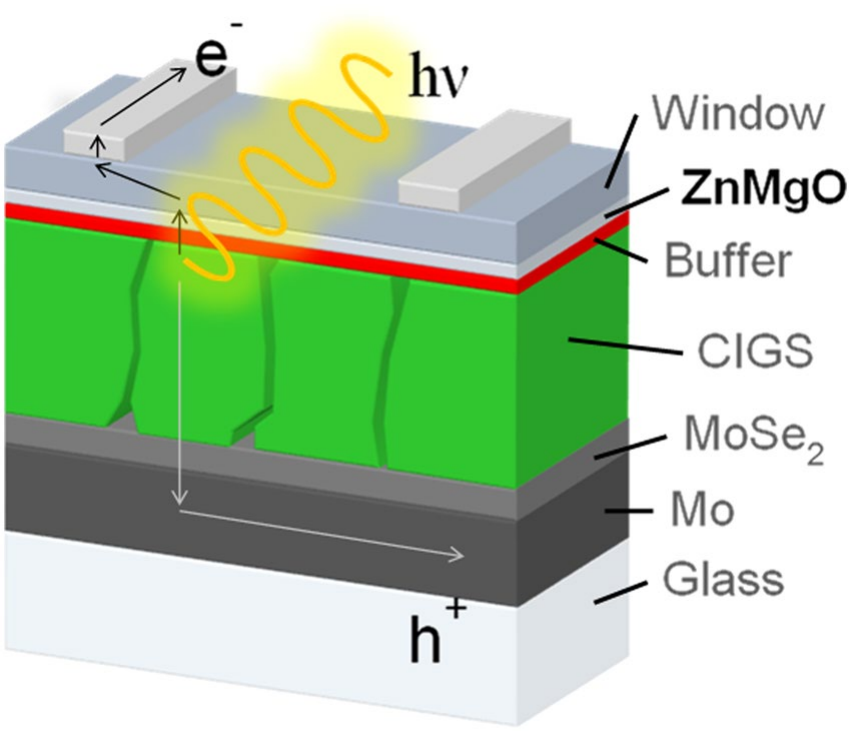
Schematic representation of a high efficiency Cd-free chalcogenide solar cell, including a back Mo contact deposited on the substrate (typically SLG), the CIGS absorber layer, a nanometric Zn(O,S) buffer layer, a ZnO based window layer and a nanometric ZnMgO interficial layer between the buffer and window ones. For high efficiency pure sulfide CIGS devices, the ZnMgO layer replaces the Zn(O,S) buffer layer.

Thin film photovoltaics have made important progress in the recent years and have recently surpassed the efficiency of multicrystalline wafer-based devices^1^. Highest efficiencies of up to 22.6% are obtained for chalcogenide solar cells based on Cu(In,Ga)(S,Se)_2_ (CIGS) chalcopyrites^2^. One of the key elements to the success of these technologies is the exploitation of the advantages of solid solution systems that by a careful variation of the composition allow achieving a fine tuning of the optoelectronic properties of the multilayer stack constituting the devices (corresponding to the device architecture schematically shown in Fig. 1). Most prominent examples are the interchange of In-Ga^3^ or S-Se^4^ to induce beneficial band-gap grading in the absorber or the use of nanometric layers with well controlled thickness and compositions to adjust the band gap and band alignment at the heterojunction^5^. In this sense, a critical feature for the development of high efficiency devices is the inclusion of a ZnMgO solid solution nanometric layer in combination with a CdS^2^ or Zn(O,S) buffer layer alternative to the standard CdS buffer layer used in these technologies^6^. Development of Cd-free high efficiency devices has a strong interest to avoid the use in these processes of heavy metals with very high toxicity as Cd. In addition, ZnMgO solid solution nanolayers are also especially promising as alternative buffer layer for wide-gap absorbers with band-gaps above 1.4 eV, as for these absorbers the conduction band alignment to the standard CdS is expected to produce a cliff. In this line, Hiroi *et al*. have recently reported a new world efficiency record for purely sulphur-based CIGS devices (E_g_ = 1.5 eV) based on the utilization of optimized ZnMgO buffer layers^7^.

While the utilization of solid solutions for these specialised nanolayers allows to precisely adjust the composition for the specific need, this also calls for the precise control of the thickness and composition of the deposited layers. This is especially the case in view of an industrialisation of these technologies on large areas, where the control of the homogeneity of thickness and composition are extremely important for the overall performance of completed solar modules with dimensions of square meters. It is therefore clear that a fast (with measuring times below 1 minute) and non-destructive methodology capable of probing thickness and composition in-line is a very valuable tool for the potential transfer of these technologies to industrial production processes.

For these applications, optical spectroscopy based on Raman scattering has already proved its versatility for controlling various important material parameters of the stacks used in thin film solar cells, including crystallinity^8^, absorber and buffer composition^9, 10^, buffer layer thickness^8^, presence of secondary interfacial phases^11, 12^ and doping concentration^13^ in separate layers, as well as complete devices^8^. This includes also the use of resonant Raman strategies for the high sensitivity assessment of nanometric layers and interfacial regions in the device structure. Resonant excitation conditions are achieved when using an excitation energy that is close to an electronic transition in the material as the energy band gap in the case of semiconductors. This coupling leads to a strong enhancement of the intensity of the Raman peaks, mainly in the case of those corresponding to LO modes, and this allows the high sensitivity detection of extremely thin (nanometric) layers in the device.

At room temperature, the most stable form of ZnO has a wurtzite crystalline structure with a direct band gap of ~3.3 eV^14^ and four active Raman modes^14, 15^, being the LO peak (that includes the contributions from the A_1_(LO) and E_1_(LO) vibrational modes) located at ~580 cm^−1^ the dominant one in the Raman spectra measured under UV resonant excitation conditions^16^. On the other hand, the most stable phase of MgO has a rocksalt structure with an extremely large band gap of ~7.8 eV^17^. In this structure the inversion symmetry at each ion site in the crystal determines the absence of first order Raman active modes^17^. The structural differences existing between the two end points of the ZnMgO solid solution lead to the existence of different crystalline structures for the solutions with Zn-rich and Mg-rich compositions, corresponding to the wurtzite and rocksalt structures, respectively. This is also accompanied by an intermediate region -corresponding to values of the relative Mg content in the range 0.33 < Mg/(Mg + Zn) < 0.45 for samples grown by reactive electron beam evaporation^18, 19^ and in the range 0.42 < Mg/(Mg + Zn) < 0.64 for samples grown by magnetron sputtering^20^- that is characterized by the existence of a mixed phase. For photovoltaic applications, the range of compositions is confined within the Zn-rich region with 0 < Mg/(Mg + Zn) < 0.33^21, 22^, where the ZnMgO solid solution has a wurtzite structure.

In this paper, we explore the use of Raman scattering as a contactless non-destructive tool for the quantitative assessment of the thickness and composition of the ZnMgO nanometric layers applied in high efficiency chalcopyrite solar cells. This has involved a detailed study of the vibrational properties of the ZnMgO solid solution nanolayers under resonant excitation conditions achieved with an excitation energy lower or higher than the energy band-gap, and a special care has been given to the analysis of the potential presence of changes in the resonant excitation conditions that can be induced by quantum confinement effects affecting the energy band gap of the studied nanolayers. Even if the study has been focused in the analysis of layers with the thickness and composition regions required for high efficiency photovoltaic applications (including layers with 0 < Zn/(Mg + Zn) < 0.33 and thicknesses typically in the range between 15 nm and 100 nm), it is interesting to remark that the proposed techniques for the quantitative assessment of the ZnMgO nanolayers are also relevant for other kinds of advanced devices involving the ZnMgO solid solution system, as UV photodetectors, light emitters and thin film transistors^23–26^.

## Materials and Methods

ZnMgO layers with different Mg contents and thicknesses were deposited by RF magnetron sputtering onto different substrates including soda lime glass (SLG), Mo coated SLG samples and different multilayered structures involving the CIGS absorber layer and the Zn(O,S) buffer layer included in the device architecture of a high efficiency CIGS solar cell, that is schematically shown in Fig. 1. The deposition was performed in a von Ardenne CS 730S (static deposition) or a Leybold in-line sputtering system (dynamic deposition) using sintered ceramic ZnMgO targets with different Mg concentrations^21^. The thickness and composition of the different layers in these samples were selected according to those used in the baseline CIGS high efficiency process established by ZSW.

The study has involved three different series of ZnMgO layers: Series (i) was formed by 5 layers that were grown with different Mg/(Mg + Zn) relative contents (in the range between 0% and 31%) and with a thickness in the range of 500–700 nm; Series (ii) included 5 layers that were grown with a constant relative Mg/(Mg + Zn) content of 10% and different thicknesses between 15 nm and 450 nm; and Series (iii) included 7 layers that were grown with a constant relative Mg/(Mg + Zn) content of 26% and different thicknesses between 19 nm and 237 nm. In all the cases, for a given composition and thickness the deposition of the layers on the different kinds of samples was made in the same sputtering deposition process to minimize potential differences between the layers grown on the different samples.

Layers series (i) were prepared to investigate the sensitivity of the Raman spectra to the Mg content. In this study, the compositions analysed were selected according to the composition region required for high efficiency chalcogenide solar cells, which corresponds to relative Mg/(Mg + Zn) contents typically in the range between 10% and 30% (depending on the composition of the absorber and buffer subjacent layers). On the other hand, layers series (ii) and (iii) were grown to deepen in the analysis of the dependence of the Raman spectra on the thickness of the ZnMgO nanolayer for compositions leading to an energy band-gap lower (series (ii)) or higher (series (iii)) than the excitation energy. In the study a special emphasis has been given to the analysis of thicknesses up to the order of 150 nm, which corresponds to the region of thicknesses that are required for these layers in high efficiency solar cells, with typical values between 15 nm and 100 nm, and layers with higher thicknesses have also been grown to include in the study samples free of quantum confinement effects.

For all the layers, the Mg content was assessed by X-Ray Photoelectron Spectroscopy (XPS). XPS experiments were performed in a PHI 5500 Multitechnique System (from Physical Electronics) with a monochromatic X-ray source (Aluminum Kα line of 1486.6 eV energy and 350 W), placed perpendicularly to the analyser axis and calibrated using the 3d_5/2_ line of Ag with full width at half maximum of 0.8 eV. The quantitative analysis has been done using the PHI Multipak 8.0 data analysis software with the sensitivity factors (SF) provided by the company, corrected and optimised for the lenses and the geometrical configuration used. SF values have been verified by the measurement of own calibrated standards.

The layer thickness was estimated using the transmission spectra measured from the layers deposited on glass. The spectra were measured using a Perkin Elmer UV-VIS Lambda 900 spectrophotometer and analysed using the software “Diplot”. This software also allows to estimate the band gap of the samples, in addition to the layer thickness.

The Raman spectra were excited with a He-Cd laser (325 nm wavelength line) and recorded using a FHR640 Horiba Jobin-Yvon spectrometer coupled with a CCD detector (Synapse from Horiba Jobin-Yvon). The measurements were performed in backscattering configuration using a Raman probe that has been developed at IREC. Focusing of the laser spot on the sample surface was made with a super long working distance objective with 20x magnification (Olympus SLMPLN 20X), which lead to an excitation laser spot diameter of ~50 µm. This has allowed to keep the excitation power density below 10 W/cm^2^, avoiding the presence of thermal effects in the spectra. This spot allows also to avoid potential effects related to microscopic in-homogeneities in the deposited layers that could compromise the interpretation of the spectra. Integration time for the measurement of each of the spectra has ranged between 5 seconds (for samples with composition Mg/(Mg + Zn) ≤ 10%) and 50 seconds (for samples with composition Mg/(Mg + Zn) ≥ 26%). The Raman shift of the spectra was calibrated using intercalated spectra obtained from the Si monocrystal reference sample and imposing its main band position at 520 cm^−1^.

## Results and Discussions

**Figure 2:**
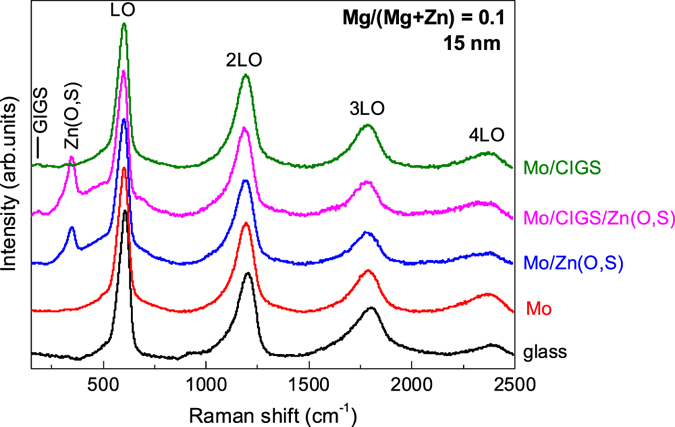
Raman scattering spectra (normalized to the intensity of the main LO peak) of ZnMgO layers grown with a relative Mg/(Mg + Zn) content of 10% and a thickness of 15 nm on the different kinds of substrates.

Figure 2 shows the Raman spectra measured on ZnMgO layers that were grown on the different samples with a composition of Mg/(Mg + Zn) = 10% and a thickness of 15 nm. As shown in the figure, the use of UV resonant excitation conditions allows the measurement of spectra with a suitable signal to noise ratio (SNR), in spite of the nanometric thickness of the layers. Interestingly, no differences are observed in the ZnMgO Raman peaks from the layers grown on the different samples. This corroborates the absence of significant differences between layers grown on the different kinds of substrates. The spectra are characterized by the presence of a dominant peak that has been identified with the main LO mode of the ZnMgO alloy, together with peaks corresponding to the 2^nd^, 3^rd^ and 4^th^ orders of this mode. Presence of these higher order peaks is strongly favoured by the use of close to resonant excitation conditions. In addition, peaks from the CIGS and Zn(O,S) underlying layers below the ZnMgO one are also observed for the layers grown on SLG/Mo/CIGS, SLG/Mo/Zn(O,S) and SLG/Mo/CIGS/Zn(O,S) samples.

### Dependence of Raman spectra on Mg/(Mg + Zn) relative content

**Figure 3:**
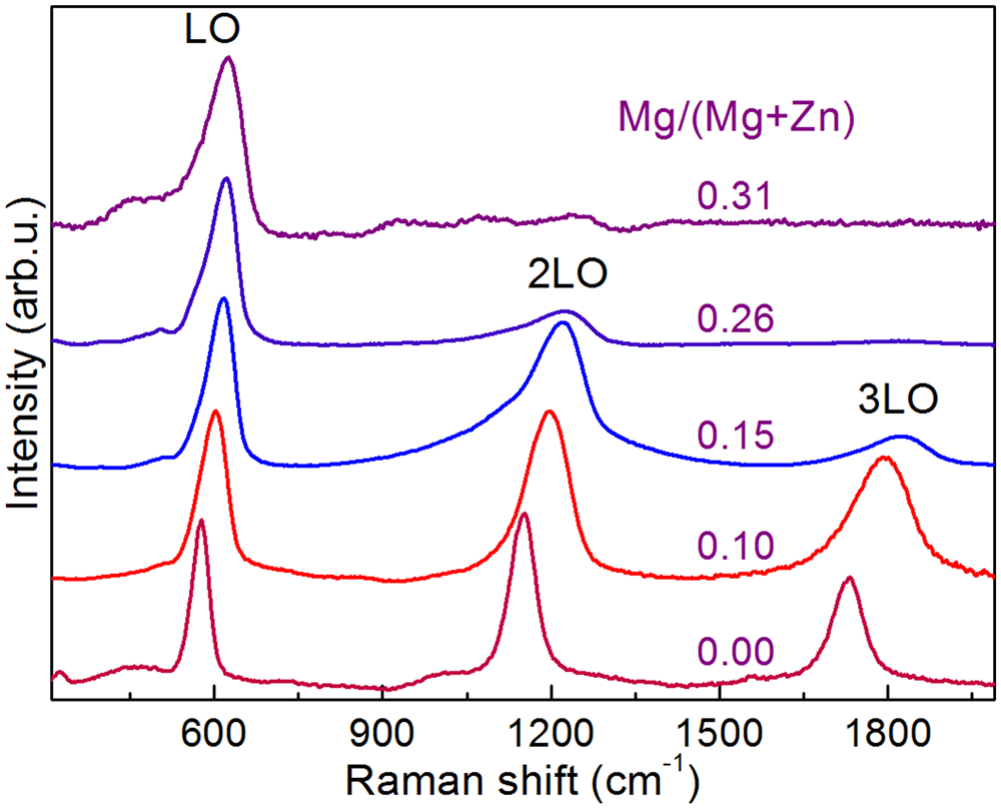
Raman scattering spectra (normalized to the intensity of the main LO peak) measured from layers series (i) that were grown with different Mg concentration.

The Raman scattering spectra measured from the layers series (i) with different Mg contents are illustrated in Fig. 3. The peaks around 600 cm^−1^, 1200 cm^−1^ and 1800 cm^−1^ have been identified as the first, second and third order of the LO vibrational mode from the ZnMgO alloy, respectively. Some weaker contributions at lower wavenumber of the fundamental and higher orders of the LO peak can also be seen. These are attributed to multi-phonon peaks, which have also been previously observed in doped ZnO layers^13, 27^. Their appearance is likely related to the presence of lattice defects^27^. The LO peaks are also characterized by an asymmetric shape towards the lower frequency side, and this asymmetry increases with the Mg content in the alloy. This behaviour has been related to the presence of disorder effects leading to a relaxation of the selection rule at q = 0 and the activation of LO phonons with larger wavevector in the Brillouin zone, in agreement with refs 28 and 29.

**Figure 4:**
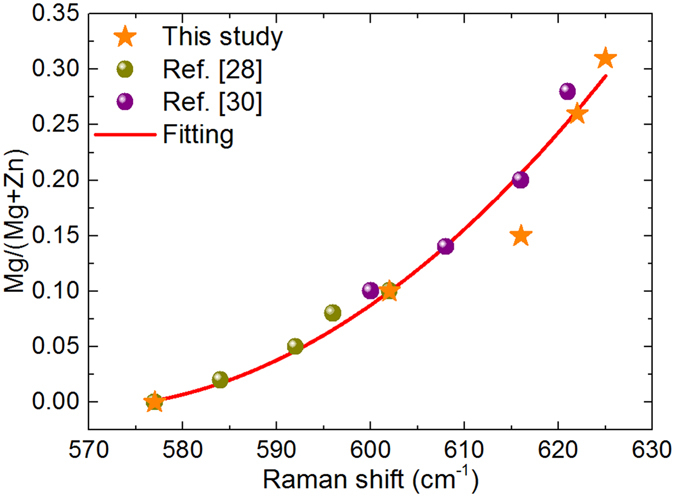
Mg/(Mg + Zn) relative content versus frequency of the main LO Raman peak. Starts: experimental data from layers series (i). Circles: data reported from references^28, 30^. Full line shows the fitting of the data according to Equation 1.

Figure 4 plots the relative Mg/(Mg + Zn) content ratio measured in these layers versus the frequency of the main LO peak in the Raman spectra. The figure includes also data previously reported in the literature^28, 30^, together with those derived from the analysis of the spectra shown in Fig. 3. All these data show the existence of a monotonous increase of the frequency of the LO mode with the Mg content in the alloy, which is related to the increase of the lattice constant with the Mg content. The dispersion between the different measurements in this figure is likely related to additional effects affecting the frequency of the Raman peaks as those induced by the presence of structural defects and/or strain. However, in spite of this, it is interesting to remark the good level of agreement of the majority of the experimental data, with only one point that shows a higher deviation. This confirms the existence of a main dependence of the frequency of the LO Raman peak on the Mg content in the solid solution for the investigated composition range.

The existence of a monotonous increase of the frequency of the LO peak with the Mg content has already been reported by Wei *et al*.^30^ and Ye *et al*.^28^ from the analysis of samples covering different ranges of compositions of the ZnMgO solid solution. These authors proposed linear empirical equations^30^ or used a modified random element isodisplacement (MREI) model with a small bowing coefficient^28^. In contrast with the linear equation proposed in ref. 30, the data plotted in Fig. 4 confirm the existence of a clear bowing in the range of compositions 0 ≤ Mg/(Mg + Zn) ≤ 33%. The presence of this bowing could be related to the reduction of the effective mass of the solid solution.

Figure 4 also shows the fitting of the experimental data with the following parabolic equation:

1 $$\frac{{\rm{Mg}}}{{\rm{Mg}}+{\rm{Zn}}}=30.33-1.07\,\times {10}^{-1}\,{\rm{\omega }}+9.39\times {10}^{-5}\,{{\rm{\omega }}}^{{\rm{2}}}.$$

In Eq. (1), ω is the wavenumber of the LO peak in cm^−1^. This equation provides with a simple empirical procedure for the assessment of the composition of the ZnMgO layers. The error estimated from the standard deviation of the difference between the experimental data and the composition given by equation (1) gives a value of ε = 0.015 in the determination of the Mg/(Mg + Zn) relative content. This corroborates the good level of agreement between the majority of experimental points and the simple equation (1). Only for the sample grown with a Mg/(Zn + Mg) relative composition of 15% there is a higher deviation. This suggests the existence in this sample of a higher contribution of stress and/or disorder effects affecting the frequency of the Raman peak.

An alternative approach for the analysis of these data is the development of a MREI model as reported in ref. 28. However, the best conditions for the application of this model require the knowledge of the frequency of the LO peak from both pure ZnO and MgO compounds. In the case of ZnO, the value of the frequency of the LO peak is well known in the literature. However, for MgO this is not so clear, because this depends of the crystalline structure of the compound. IR spectroscopy gives values of 725 cm^−1^ ^31^ or 727 cm^−1^ ^32^ for the LO peak in rocksalt MgO. However, the frequency of the peak could differ substantially for a wurtzite MgO. In this case in ref. 28 the authors obtained a value of 662 cm^−1^ for the LO peak from wurtzite MgO. In these conditions, use of a parabolic empirical equation based in the fitting of the data as proposed in this work provides with a simple methodology for quantification of the composition. A more detailed quantification with lower uncertainty implies the need for a higher number of reference samples with well calibrated compositions. In addition, special care has also to be taken into account when applying these empirical equations. In this sense, the data reported in ref. 29 show deviations significantly higher than the reported uncertainty of ε = 0.015 in relation to the empirical equation (1), which could be related to the different processes and measuring conditions used in this work.

### Raman scattering assessment of the ZnMgO layer thickness

Several approaches have been studied in this work for the quantitative Raman scattering assessment of the thickness of the nanometric ZnMgO layers. These have included: (a) the analysis of the intensity of the ZnMgO main Raman peak; (b) the evaluation of the changes of the intensity of the main Raman peak from the subjacent layer located below the ZnMgO one; and (c) the study of changes in the relative intensity of the first to higher order ZnMgO peaks. In principle, the two first approaches are directly related to the thickness of the layer under analysis, while the third one constitutes an indirect approach that is based in the existence of a correlation between the nanometric size of the grains in the layers and their thickness. However, this correlation implies also the existence of potential changes in the band gap of the ZnMgO layers with the thickness of the layers that are determined by the presence of quantum confinement effects in the nanometric grains. Changes in the band gap are relevant for the interpretation of the intensity of the Raman peaks from the spectra because they determine also changes in the resonant excitation conditions used for the measurements performed in the different layers. As will be shown in next sections, these features are strongly determining for the evaluation of the thickness of the ZnMgO layers.

#### Sensitivity of the intensity of the ZnMgO Raman peaks to changes of the layer thickness

Analysis of the changes in the intensity of the main Raman peak from the deposited layer has already been reported for the non-destructive assessment of the thickness of nanometric CdS buffer layers that are currently used in chalcogenide solar cells, and the systematic detection of the changes in the intensity of the peak has been applied for the detection of buffer layer in-homogenities that can determine efficiency loses in large area solar modules^8, 33^. In these cases, the analysis is based in the existence of a direct correlation between the intensity of the main Raman peak and the thickness of the layer. This is related to the dependence of the intensity of the CdS peaks on the CdS volume fraction included in the total volume contributing to the scattering of the photons in the Raman spectra. Accordingly, this correlation occurs in the range of thicknesses where the photons from the excitation laser are able to penetrate through the whole layer thickness.

**Figure 5:**
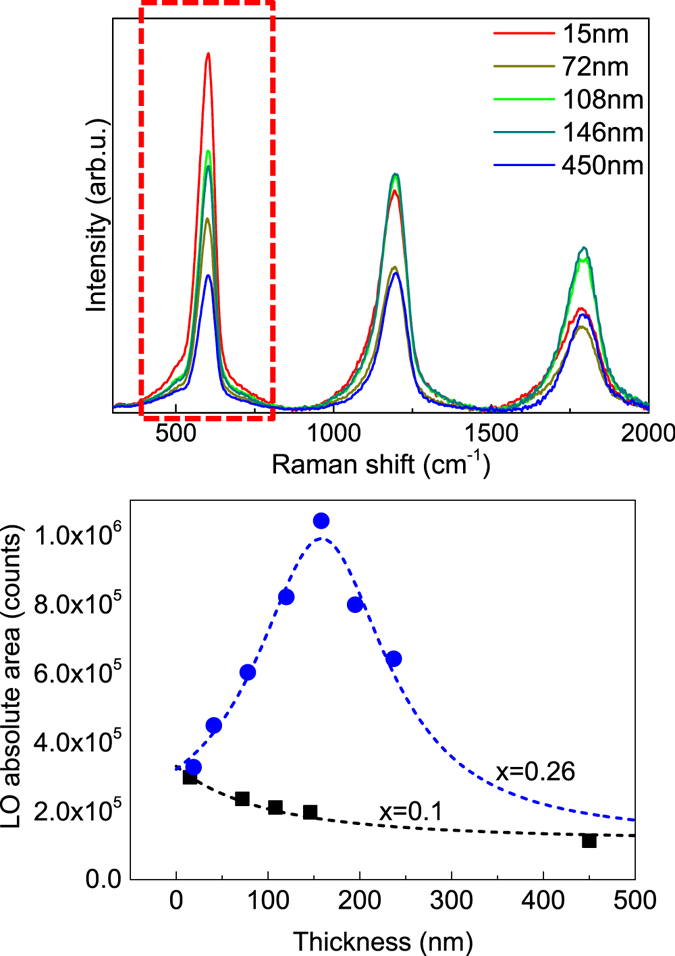
Top: Raman spectra measured from series (ii) layers with Mg/(Mg + Zn) relative content 10% and different layer thicknesses. Bottom: Integral intensity of the main LO peak (highlighted in red in the Raman spectra plotted in Fig. 4 top) versus layer thickness for layers series (ii) (Mg/(Mg + Zn) relative content 10%) and layers series (iii) (Mg/(Mg + Zn) relative content 26%). Dotted lines are added as guide for the eye.

Figure 5 shows the plot of the intensity of the main ZnMgO LO peak versus the thickness of the layers for the layers grown with Mg/(Mg + Zn) relative contents of 10% (series (ii)) and 26% (series (iii)). In both cases the intensity of the LO peak shows a strong dependence on the layer thickness, which corroborates the potential of this strategy for the quantification of changes in the layer thickness. However, in this case there are strong differences in the observed behaviour from the different series of samples. For samples series (ii) the intensity of the peak shows a monotonous decrease when the layer thickness increases, while for samples series (iii) the intensity of the peak has a well-defined maximum for a thickness of 158 nm, and the intensity strongly decreases when the thickness goes away from this value.

This behaviour is directly related to the existence of a correlation between the grain size in the nanometric layers and the layer thickness, which determines changes in the band gap of the layers grown with different thicknesses due to the presence of quantum confinement effects. Accordingly, decreasing the layer thickness leads to a corresponding decrease in the grain size which in turns determines an increase in the energy band gap in relation to the values estimated for the solid solution alloy free of quantum confinement effects. According to^20^, these correspond to values of ~ 3.51 eV and ~3.74 eV for the samples with relative Mg/(Mg + Zn) contents of 10% and 26%, respectively. The experimental data plotted in Fig. 5 point out the existence of two different behaviours, depending on the layer composition:

- Layers series (ii): In this case the energy of the excitation 325 nm laser line (3.8 eV) is higher than the band gap of the layers for the whole range of layer thicknesses. Increasing the layer thickness leads to a decrease of the energy band gap towards the estimated value of ~3.51 eV, and this determines a gradual loosening of the resonant excitation conditions which leads to the observed decrease in the intensity of the peak in spite of the higher thickness of the layers.
- Layers series (iii): In this case the energy of the excitation 325 nm laser line (3.8 eV) is lower than the band gap of the layers grown with lower thicknesses. As in the previous case, increasing the layer thickness leads to a decrease of the energy band gap but in this case this determines a gradual increase of the resonant excitation conditions. This explains the strong increase observed in the intensity of the Raman peak when the thickness of the layers increases up to 158 nm, where a resonant peak is observed in the spectra. This agrees with the band gap values experimentally estimated from the optical transmission measurements performed on the layers from this series grown on glass substrates, which show a decrease of the band gap from a value of 3.895 eV (for the thinnest 19 nm layer) to a value of 3.834 eV corresponding to the resonant peak observed in the sample with a thickness of 158 nm. Further increase of the thickness leads to a further decrease of the band gap towards the value of 3.74 eV estimated for this solid solution and, in turn, to a decrease of the intensity of the Raman peaks of the layer.

These measurements provide with a direct experimental evidence on the existence of a correlation between the thickness of these nanometric layers and the grain size that leads to the observed changes in the band gap of the layers. For the experimental conditions used in this work, these measurements are sensitive to changes in the thickness of the layers of the order of ≤2.5 nm for layers with thicknesses in the 15–30 nm region. This corroborates the potential of these measurements for the high sensitivity detection of nanometric changes in the thickness of the layers. Calibration of these effects for the quantitative assessment of the thickness of the layers implies the need for reference layers with a constant composition corresponding to the targeted one leading to optimal device performance. On the other hand, the assessment of the composition of the layers from the analysis of the frequency of the LO peak and according to equation 1 would allow excluding the potential existence of changes in the composition of the layers that could lead to misinterpretation of the experimental data.

The non-monotonous change of the LO absolute area versus the layer thickness observed in Fig. 5 for the samples with higher Mg content conditions the applicability of this method for the assessment of the thickness of layers with this range of compositions. As shown in the figure, the LO Raman peaks from layers with a Mg/(Mg + Zn) relative content of x = 0.26 and thicknesses of 120 nm and 195 nm have a very similar value of the area. In this case the right interpretation of these data requires a previous knowledge on the range of the layer thickness (higher or lower than 158 nm). For the used 325 nm excitation wavelength, this behaviour takes place for Mg/(Mg + Zn) relative contents higher than x = 0.20, and lower Mg contents lead to the appearance of a monotonous decrease of the Raman peak intensity when the layer thickness increases. Increasing the excitation laser energy would lead to an increase of the Mg content region leading to a monotonous behaviour of the intensity of the LO Raman peak with the layer thickness.

#### Sensitivity of the intensity of the main Raman peak from the subjacent region below the ZnMgO layer to changes of the layer thickness

**Figure 6:**
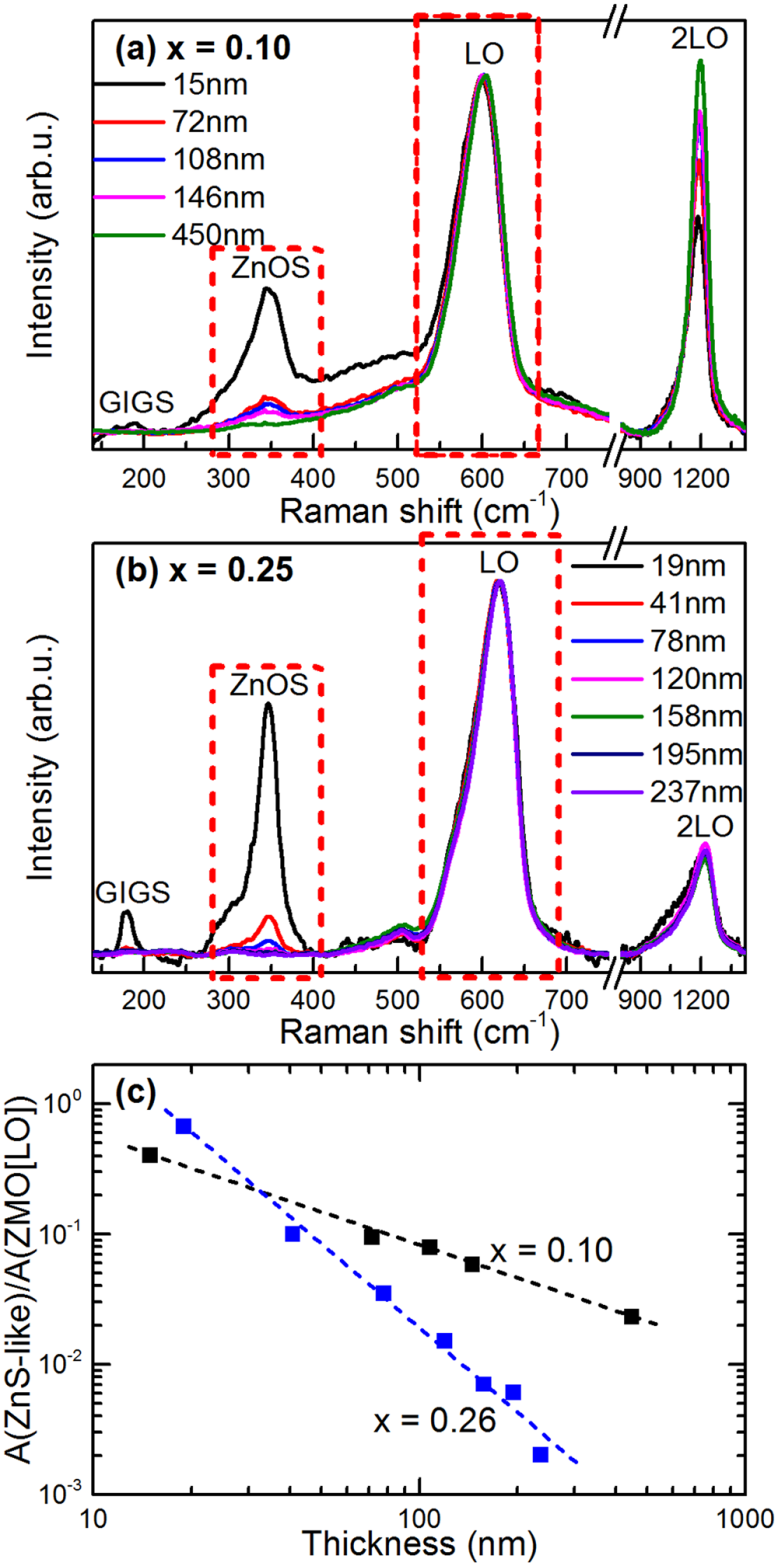
Raman spectra (normalised to the intensity of the main LO peak) measured from series (ii–**a**) and (iii–**b**) layers. (**c**) Relative intensity of the ZnS-like peak from the Zn(O,S) subjacent layer in relation to the main LO ZnMgO Raman peak (corresponding to the peaks highlighted in red in the Raman spectra) versus the layer thickness for layers series (ii) (Mg/(Mg + Zn) relative content 10%) and layers series (iii) (Mg/(Mg + Zn) relative content 26%). Dotted lines correspond to the fitting of the data with linear relationships in the log-log scale.

In order to analyse the dependence of the intensity of the Raman peaks from the region located below the ZnMgO layer on the layer thickness we have selected the layers that were grown on SLG/glass/Mo/CIGS/Zn(O,S) samples, and the analysis has been centred on the study of the main Raman peak from the Zn(O,S) buffer layer in the device heterostructure which corresponds to the ZnS-like peak from the Zn(O,S) alloy^10^. As can be seen in Fig. 6, an increase in the thickness of the ZnMgO layer results in a decrease in the relative intensity of the ZnS-like peak of the underlying Zn(O,S) buffer layer, in relation to the intensity of the main ZnMgO LO peak. A similar approach has been reported in the literature for the non-destructive characterization of the uniformity of thin cobalt disilicide films deposited on Si substrates^34^. An increase in the thickness of the top layer results in an increase in the absorption of the excitation light by the layer, which causes a decrease in the interaction between the excitation laser and the region located below the layer.

Figure 6 shows the plot of the relative intensity of the main Zn(O,S) Raman peak in relation to that of the LO ZnMgO peak versus the thickness of the layers for layers grown with Mg/(Mg + Zn) relative contents of 10% (series (ii)) and 26% (series (iii)). In both cases, the intensity of the ZnS-like peak of the Zn(O,S) buffer layer shows a linear dependence on the layer thickness when using a log-log plot.

These experimental data corroborate the viability of these measurements for the quantitative estimation of the thickness of the ZnMgO layer for layer thicknesses up to 150 nm. For higher thicknesses, the layer becomes too thick and this prevents detection of the Raman peaks from the subjacent region. Nonetheless, the slope of the fitting line depends on the Mg content. This is due to the dependence of this slope on the absorption coefficient of the ZnMgO layer that, in turn, is determined by the layer composition. Accordingly, and similarly to the previous case, the calibration of these curves has to be performed using reference samples grown with a constant composition corresponding to the desired ZnMgO layer composition in the optimized devices. For the experimental conditions used in this work, these measurements are sensitive to changes in the thickness of the layers of the order of ≤2 nm, for layers with thicknesses in the 15–30 nm region. As in the previous case, correlation of these measurements with the assessment of the composition of the layers from the analysis of the frequency of the main LO ZnMgO peak allows excluding the potential presence of effects related to changes in the Mg content in the layer.

#### Sensitivity of the relation between the intensity of the first and second/third order LO ZnMgO Raman peaks to changes of the layer thickness

In addition to the above mentioned effects, the relative intensity of the 2^nd^/3^rd^ order peaks in relation to the fundamental LO Raman peak from the ZnMgO solid solution is also sensitive to changes of the layer thickness, because of the correlation observed in these samples between the layer thickness and the grain size of the nanocrystals in the layers. Variations in the relative intensity of the first order peak (LO) in relation to that of the higher order peaks (2LO, 3LO, etc.) have been previously observed in ZnS thin films^35^, ZnO quantum dots^36^ and nanowires^37^, ZnTe nanoparticles^38^, as well as in other materials.

**Figure 7:**
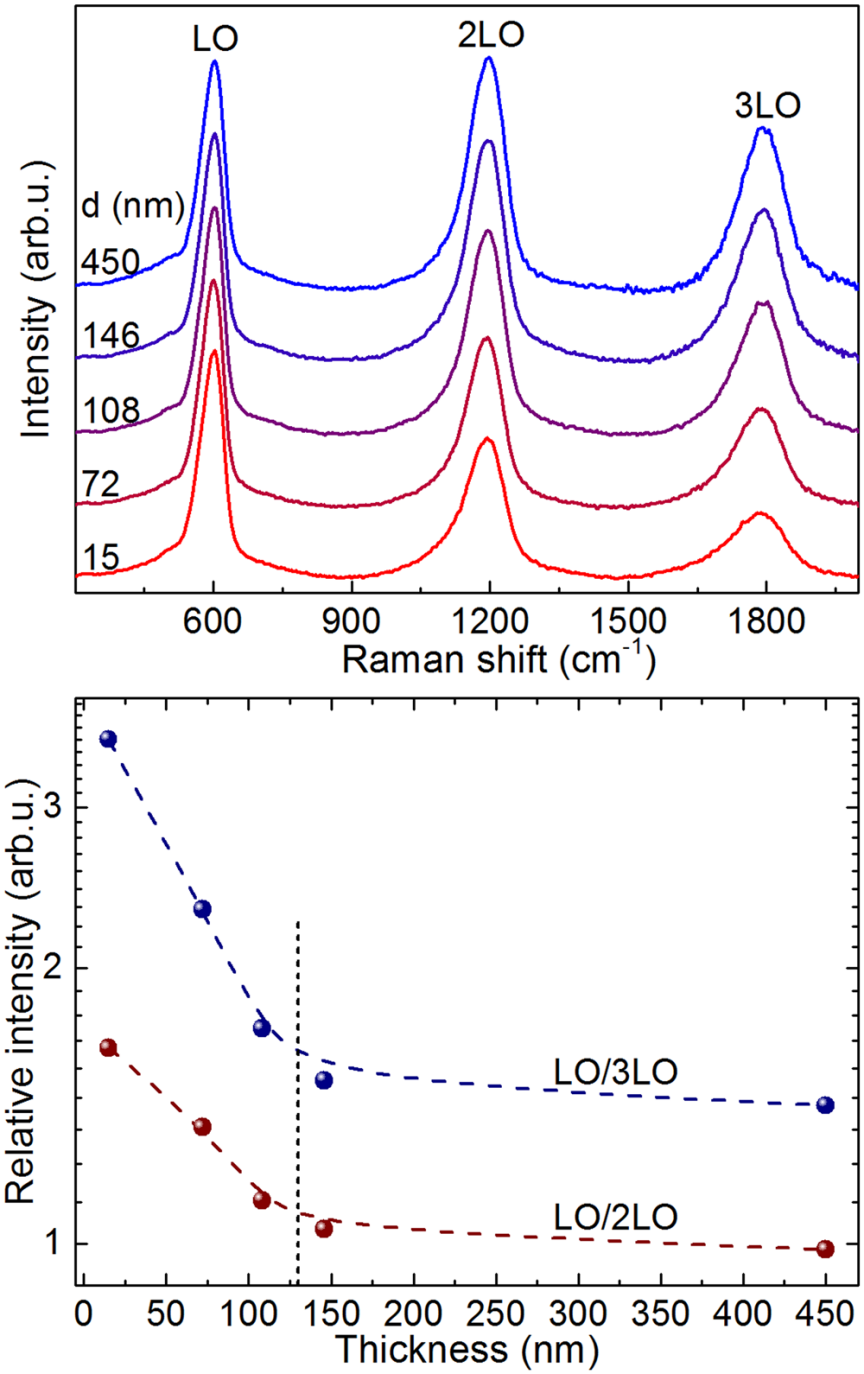
Top: Raman spectra (normalized to the intensity of the LO peak) from layers series (ii) with Mg/(Mg + Zn) = 0.1 and different thickness. Bottom: Relative intensity of the first order LO peak in relation to that of the second order and third order peaks versus layer thickness.

The Raman scattering spectra measured for the layers series (ii) with Mg/(Mg + Zn) = 0.1 and different thicknesses are shown in Fig. 7. An analysis of the spectra clearly shows that the intensity of the LO peak relative to the 2LO and 3LO peaks depends on the thickness of the layers. Increasing the thickness up to about 150 nm leads to a significant decrease in the relative intensity of the LO peak in relation to that of the higher order peaks. For layers with a thickness higher than 150 nm, the relative intensity of the peak remains almost constant. This is related to the changes in the electron-phonon interaction determined by the quantum confinement effects taking place in the nanocrystalline layers grown with these thicknesses.

This behaviour provides with an alternative approach for the non-destructive assessment of the thickness of the layers in the range of thicknesses of interest for the development of high efficiency chalcogenide solar cells. The analysis of the experimental data shown in Fig. 7 gives a lower sensitivity of this method to the changes of thickness of the layers than those described in the previous sections, being in this case the measurements sensitive to thickness changes down to ≤6 nm, for the used experimental conditions and for layers with thicknesses in the 15–30 nm region. A potential advantage of this method in relation to the previous approaches is that this method does not require the presence of additional Raman peaks from the subjacent region below the layer. In addition, the use of a relative intensity approach for the analysis of the data has advantages because of their higher tolerance to potential changes in the intensity of the excitation light during the measurements that could be related either to instabilities of the laser source or to changes in the focusing of the laser spot on the sample surface, for example.

However, also in this case the presence of these effects is strongly dependent on the composition of the layers: For the layers series (iii) with Mg/(Mg + Zn) = 0.26 these effects have not been observed and the relative intensity of the 1^st^ order LO peak does not show changes in relation to those from the higher order peaks. This has been related to the use in these measurements of an excitation energy that is lower that the band gap of the ZnMgO nanolayers as discussed above.

The differences observed between the behaviour of the intensity of the first and higher order Raman peaks when using an excitation energy higher or lower than the energy band-gap are likely related to the differences in the interaction between the phonons and the bound excitons. This interaction has been observed from Photoluminescence measurements performed in ZnMgO solid solutions^39, 40^. The interaction between phonons and bound excitons has also been reported in ZnTe^38^ to explain the observation of different temperatures where the relative intensity of the 2LO and 3LO peaks has a maximum value. In the case of the ZnMgO layers with Mg/(Mg + Zn) = 10% and thicknesses lower than about 150 nm, the quantum confinement effect also influences the bound exciton energy, that shifts to higher values when the thickness decreases. This determines a change in the photon to bound exciton interaction which is likely responsible for the changes observed in the relative intensity of the fundamental LO mode in relation to that of the higher order peaks. In the case of the nanolayers with Mg/(Mg + Zn) = 0.26 the energy band gap is higher than the excitation laser energy. Under these conditions it is not possible to excite any bound exciton in the material, and therefore the relative intensity of the different order peaks in the Raman spectra is not affected. Hence, the changes in the intensity of the different peaks in the spectra are only determined by the proximity of the excitation energy to the resonant excitation conditions and are equal for all peaks (LO, 2LO and 3LO).

## Conclusions

The detailed vibrational analysis of nanometric ZnMgO layers grown with different compositions and thicknesses has allowed deepening in the knowledge of the sensitivity of the different spectral features of the Raman spectra on the characteristics of the layers. The obtained results corroborate the viability of resonant Raman scattering based techniques for the non-destructive quantitative assessment of the layers with thickness and compositions corresponding to those required for the development of high efficiency chalcogenide solar cells. The study of the spectra measured on layers grown with different compositions indicates that the frequency of the LO peak is mainly affected by the Zn content in the solid solution and no relevant effects are observed related to the potential presence of stress and/or structural defects, for the studied samples. The data obtained agree well with the experimental data previously reported in refs 28 and 30. Fitting of all these data provides with a simple empirical parabolic equation that is proposed for the quantitative assessment of the composition of the solid solutions with an estimated uncertainty of ε = 0.015. The study of layers that were grown with different thicknesses has also allowed to analyse the viability of different experimental approaches for the non-destructive quantitative assessment of the thickness of the layer, including: (a) the analysis of the intensity of the ZnMgO main Raman peak; (b) the evaluation of the changes of the intensity of the main Raman peak from the subjacent layer located below the ZnMgO layer; and (c) the study of changes in the relative intensity of the first to second/third order ZnMgO peaks. In all the cases, the analysis performed has corroborated the potential of these approaches for the quantitative detection of changes in the thickness of the layers down to values in the range ≤(2 nm–6.5 nm) (depending on the approach) for layers with thicknesses in the range 15 nm–30 nm. These measurements are also strongly dependent on the layer composition, and the establishment of a quantitative methodology requires for the detailed calibration of the measurements using series of reference layers grown with the desired composition. On the other hand, in the first and third cases the behaviour observed is strongly determined by the existence of a correlation between the grain size in the nanocrystalline layers and their thickness. This determines an increase of the energy band gap of the layers when the thickness decreases, which in turns leads to gradual changes in the resonant excitation conditions achieved with the used UV excitation wavelength. This explains the strong differences found in the intensity of the peaks in the Raman spectra measured from nanolayers grown with compositions and thicknesses leading to energy band-gaps higher or lower than the excitation energy. Finally, the differences found in the changes of the relative intensity of the 1^st^, 2^nd^ and 3^rd^ order LO peaks with the layer thickness for layers grown with different compositions suggest that these effects are related to the interaction between the excitation photons and bound excitons, which explains the need to work with an excitation energy higher than the band-gap to observe these effects.
